# (*E*)-4-[7-(2,3-Di­hydro­thieno[3,4-*b*][1,4]dioxin-5-yl)-2,1,3-benzo­thia­diazol-4-yl]-2-[(neo­pentyl­imino)­meth­yl]phenol

**DOI:** 10.1107/S1600536814014883

**Published:** 2014-07-05

**Authors:** Lauren A. Mitchell, Jordan A. Dinser, Bradley J. Holliday

**Affiliations:** a Department of Chemistry, The University of Texas at Austin, 105 E 24th Street, Stop A5300, Austin, Texas 78712, USA

**Keywords:** crystal structure

## Abstract

In the title mol­ecule, C_24_H_23_N_3_O_3_S_2_, the benzo­thia­diazole ring system is essentially planar, with an r.m.s. deviation of 0.020 (8) Å. The thio­phene and hy­droxy-substitiuted rings form dihedral angles of 23.43 (9) and 35.45 (9)°, respectively, with the benzo­thia­diazole ring system. An intra­molecular O—H⋯N hydrogen bond is observed. In the crystal, weak C—H⋯O hydrogen bonds and π–π stacking inter­actions [centroid–centroid distance = 3.880 (3) Å] link mol­ecules into chains along [100]. In addition, there are short S⋯S contacts [3.532 (3) Å] which link these chains, forming a two-dimensional network parallel to (010).

## Related literature   

For related structures, see: Mejía *et al.* (2010 ▶); Wong *et al.* (2008 ▶). For the properties of 3,4-ethyl­ene­dioxy­thio­phene and benzo­thia­diazole compounds, see: Sendur *et al.* (2010 ▶); Tanriverdi *et al.* (2012 ▶); Holliday *et al.* (2006 ▶); Ellinger *et al.* (2011 ▶). For the synthesis of the starting material 5-(7-(2,3-di­hydro­thieno[3,4-*b*][1,4]dioxin-5-yl)benzo[*c*][1,2,5]thia­diazol-4-yl)-2-hy­droxy­benzaldehyde, see: Dinser (2013 ▶). For previous reports of S⋯S inter­actions, see: Chen *et al.* (2009 ▶); Reinheimer *et al.* (2009 ▶).
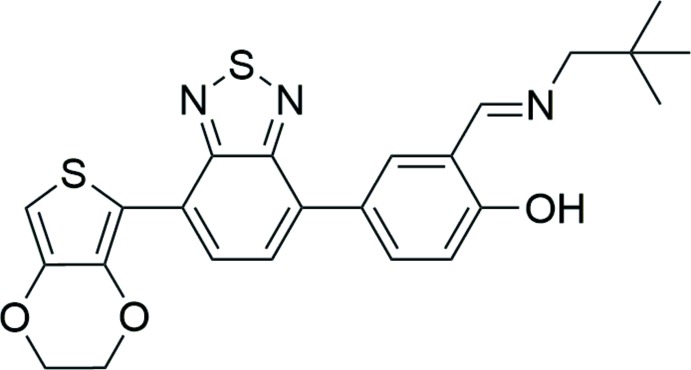


## Experimental   

### 

#### Crystal data  

C_24_H_23_N_3_O_3_S_2_
*M* *_r_* = 465.57
Triclinic, 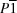

*a* = 8.040 (5) Å
*b* = 11.071 (8) Å
*c* = 12.650 (9) Å
α = 96.882 (13)°
β = 93.221 (11)°
γ = 96.065 (8)°
*V* = 1109.0 (13) Å^3^
*Z* = 2
Mo *K*α radiation
μ = 0.27 mm^−1^
*T* = 153 K
0.15 × 0.07 × 0.05 mm

#### Data collection  

Rigaku Mercury2 diffractometer
Absorption correction: multi-scan (*ABSCOR*; Higashi, 2001 ▶) *T*_min_ = 0.830, *T*_max_ = 1.000
16304 measured reflections
3899 independent reflections
2670 reflections with *I* > 2σ(*I*)
*R*_int_ = 0.100

#### Refinement  

*R*[*F* ^2^ > 2σ(*F* ^2^)] = 0.061
*wR*(*F* ^2^) = 0.168
*S* = 1.00
3899 reflections
297 parameters
H atoms treated by a mixture of independent and constrained refinement
Δρ_max_ = 0.26 e Å^−3^
Δρ_min_ = −0.30 e Å^−3^

### 

Data collection: *CrystalClear* (Rigaku, 2008 ▶); cell refinement: *CrystalClear*; data reduction: *CrystalClear*; program(s) used to solve structure: *SIR97* (Altomare *et al.*, 1999 ▶); program(s) used to refine structure: *SHELXL97* (Sheldrick, 2008 ▶) within *WinGX* (Farrugia, 2012 ▶); molecular graphics: *ORTEP-3* for Windows (Farrugia, 2012 ▶), *POV-RAY* (Cason, 2004 ▶) and *Mercury* (Macrae *et al.*, 2008 ▶); software used to prepare material for publication: *SHELXL97* and *publCIF* (Westrip, 2010 ▶).

## Supplementary Material

Crystal structure: contains datablock(s) I. DOI: 10.1107/S1600536814014883/lh5710sup1.cif

Structure factors: contains datablock(s) I. DOI: 10.1107/S1600536814014883/lh5710Isup2.hkl

Supporting information file. DOI: 10.1107/S1600536814014883/lh5710Isup3.cml

CCDC reference: 1010080

Additional supporting information:  crystallographic information; 3D view; checkCIF report

## Figures and Tables

**Table 1:** Hydrogen-bond geometry (Å, °)

*D*—H⋯*A*	*D*—H	H⋯*A*	*D*⋯*A*	*D*—H⋯*A*
O3—H16⋯N3	0.98 (6)	1.64 (7)	2.569 (4)	155 (6)
C4—H4*A*⋯O3^i^	0.97	2.39	3.200 (5)	140

Symmetry code: (i) 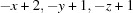.

**Fig. 1.**
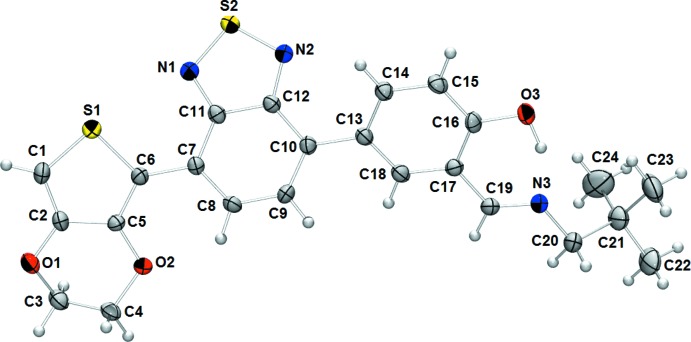
The molecular structure of the title compound. Ellipsoids are drawn at the 50% probability level.

**Fig. 2.**
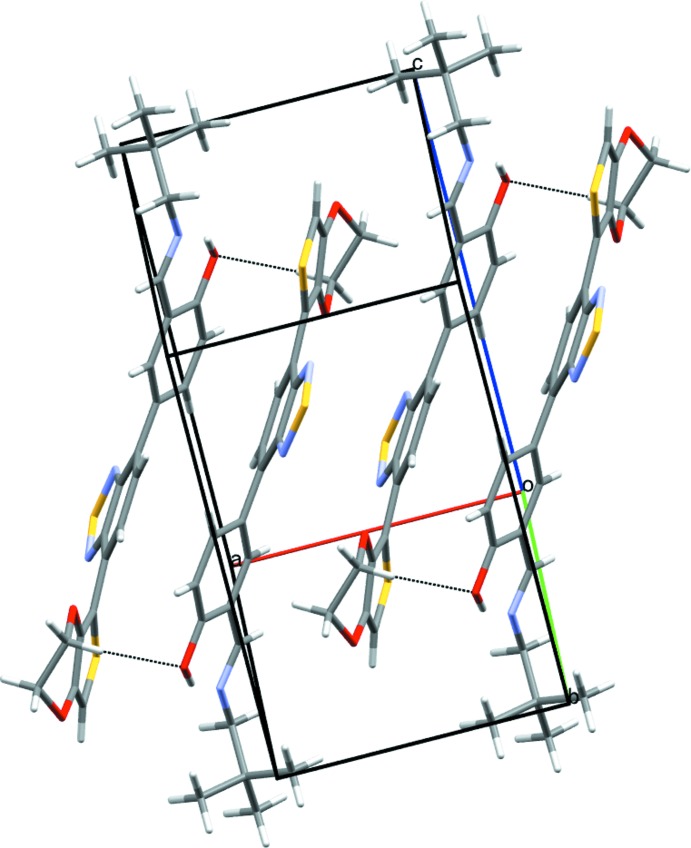
Crystal structure viewed along the b axis. Interactions are shown between O3 and H4a of neighboring molecules.

**Fig. 3.**
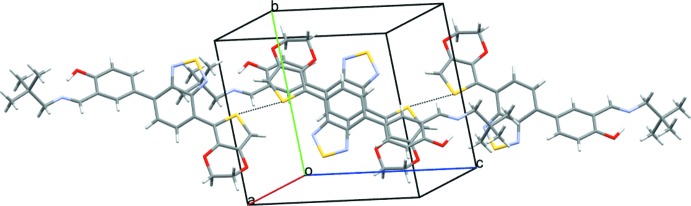
Crystal structure viewed along the a axis. Interactions are shown between S1 and S1 of neighboring molecules.

## supplementary crystallographic information

### S1. Comment

The multiple functionalities of the title molecule make it a promising material for a range of applications. Both benzothiadiazole and 3,4-ethylenedioxythiophene containing compounds have been utilized in a wide range of applications including photovoltaics (Sendur *et al.*, 2010), sensors (Tanriverdi *et al.*, 2012; Holliday *et al.*, 2006), non-linear optics and luminescent materials (Ellinger *et al.*, 2011).

The molecular structure of the title compound is shown in Fig. 1. The dihedral angle between the benzothiadiazole moeity and the thiophene ring is 23.43 (9)° and the dihedral angle between the benzothiadiazole moeity and the phenol ring is 35.45 (9)°. The geometry of the ethylenedioxythiophene moiety is similar to other ethylenedioxythiophene containing compounds reported in the literature (Mejía *et al.*, 2010; Wong *et al.*, 2008). In the crystal, weak C—H···O hydrogen bonds and π–π stacking interactions (centroid–centroid distance = 3.880 (3) Å) link the molecules into chains along [100] (Fig. 2). The π–π interactions involve inversion related rings containing atoms C7-C12. In addition, there are short S···S contacts (3.532 (3) Å) which link these chains forming a two-dimensional network parallel to (010) (Fig. 3). The S···S interactions compare to those observed perviously by Chen *et al.* (2009) and Reinheimer *et al.* (2009) which are in the range 3.396 (1) - 3.470 (1) Å and 3.580 (4) Å respectively. An intramolecular O—H···N hydrogen bond is also observed.

### S2. Experimental

The title compound was prepared by a condensation reaction between 5-(7-(2,3-dihydrothieno[3,4-*b*][1,4]dioxin-5-yl)benzo[*c*][1,2,5] thiadiazol-4-yl)-2-hydroxybenzaldehyde, prepared following Dinser (2013), and neopentylamine. The aryl aldehyde (1.41 g, 3.58 mmol) was dissolved in 120 ml of dichloromethane with the aid of sonication. To this solution was added 100 ml of ethanol followed by a concentrated solution of neopenylamine (0.24 ml, 2.05 mmol) dissolved in approximately 2 ml of ethanol. The reaction mixture was then further diluted with 98 ml of ethanol. The reaction mixture was stirred at room temperature for 5 h before the total solvent volume was reduced to approximately 100 ml by rotary evaporation at reduced pressure. Upon standing the product precipitated and was isolated by vacuum filtration. Single crystals suitable for X-ray diffraction were isolated from this sample. ^1^H NMR (400 MHz, CDCl_3_): δ 8.44 (s, 1H), 8.42 (d, 1H, *J* = 7.6 Hz), 8.03 (d, 1H, *J* = 2.4 Hz), 7.92 (dd, 1H, *J* = 2.2, 9.0 Hz), 7.71 (d, 1H, *J* = 7.6), 7.15 (d, 1H, *J* = 8.4 Hz), 6.60 (s, 1H), 4.44 (m, 2H), 4.34 (m, 2H), 3.42 (s, 1H), 1.03 (s, 9H). FTIR: ν = 1633 cm^-1^ (C=N).

### S3. Refinement

The hydroxy H atom and the H atom bonded to C19 were refined independently with isotropic displacement parameters. All other H atoms were positioned geometrically and refined using a riding-model approximation, with C—H = 0.93–0.97 Å and with *U*_iso_(H) = 1.2 times *U*_eq_(C) or *U*_iso_(H) = 1.5 times *U*_eq_(C_methyl_).

### Crystal data

**Table:**

C_24_H_23_N_3_O_3_S_2_	*Z* = 2
*M**_r_* = 465.57	*F*(000) = 488
Triclinic, *P*1	*D*_x_ = 1.394 Mg m^−^^3^
Hall symbol: -P 1	Mo *K*α radiation, λ = 0.71075 Å
*a* = 8.040 (5) Å	Cell parameters from 1661 reflections
*b* = 11.071 (8) Å	θ = 2.3–31.9°
*c* = 12.650 (9) Å	µ = 0.27 mm^−^^1^
α = 96.882 (13)°	*T* = 153 K
β = 93.221 (11)°	Prism, orange
γ = 96.065 (8)°	0.15 × 0.07 × 0.05 mm
*V* = 1109.0 (13) Å^3^	

### Data collection

**Table:**

Rigaku Mercury2 diffractometer	3899 independent reflections
Radiation source: fine-focus sealed tube	2670 reflections with *I* > 2σ(*I*)
Graphite monochromator	*R*_int_ = 0.100
Detector resolution: 13.6612 pixels mm^-1^	θ_max_ = 25.0°, θ_min_ = 2.6°
ω scans	*h* = −9→9
Absorption correction: multi-scan (*ABSCOR*; Higashi, 2001)	*k* = −13→13
*T*_min_ = 0.830, *T*_max_ = 1.000	*l* = −15→15
16304 measured reflections	

### Refinement

**Table:**

Refinement on *F*^2^	Primary atom site location: structure-invariant direct methods
Least-squares matrix: full	Secondary atom site location: difference Fourier map
*R*[*F*^2^ > 2σ(*F*^2^)] = 0.061	Hydrogen site location: inferred from neighbouring sites
*wR*(*F*^2^) = 0.168	H atoms treated by a mixture of independent and constrained refinement
*S* = 1.00	*w* = 1/[σ^2^(*F*_o_^2^) + (0.0903*P*)^2^] where *P* = (*F*_o_^2^ + 2*F*_c_^2^)/3
3899 reflections	(Δ/σ)_max_ < 0.001
297 parameters	Δρ_max_ = 0.26 e Å^−^^3^
0 restraints	Δρ_min_ = −0.30 e Å^−^^3^

### Special details

**Table:**

Geometry. All e.s.d.'s (except the e.s.d. in the dihedral angle between two l.s. planes) are estimated using the full covariance matrix. The cell e.s.d.'s are taken into account individually in the estimation of e.s.d.'s in distances, angles and torsion angles; correlations between e.s.d.'s in cell parameters are only used when they are defined by crystal symmetry. An approximate (isotropic) treatment of cell e.s.d.'s is used for estimating e.s.d.'s involving l.s. planes.
Refinement. Refinement of *F*^2^ against ALL reflections. The weighted *R*-factor *wR* and goodness of fit *S* are based on *F*^2^, conventional *R*-factors *R* are based on *F*, with *F* set to zero for negative *F*^2^. The threshold expression of *F*^2^ > σ(*F*^2^) is used only for calculating *R*-factors(gt) *etc*. and is not relevant to the choice of reflections for refinement. *R*-factors based on *F*^2^ are statistically about twice as large as those based on *F*, and *R*-factors based on ALL data will be even larger.

### Fractional atomic coordinates and isotropic or equivalent isotropic displacement parameters (Å^2^)

**Table:**

	*x*	*y*	*z*	*U*_iso_*/*U*_eq_	
S1	0.50615 (13)	0.46796 (9)	0.86018 (8)	0.0338 (3)	
S2	0.60437 (14)	0.82607 (9)	0.72634 (8)	0.0357 (3)	
O1	0.3551 (3)	0.1211 (2)	0.8028 (2)	0.0355 (7)	
O3	1.2110 (3)	0.8933 (2)	0.2468 (2)	0.0313 (6)	
N3	1.1173 (4)	0.7203 (3)	0.0944 (2)	0.0275 (7)	
N2	0.7150 (4)	0.8099 (3)	0.6241 (2)	0.0282 (7)	
N1	0.5568 (4)	0.6858 (3)	0.7444 (2)	0.0300 (8)	
C13	0.9076 (4)	0.7017 (3)	0.4432 (3)	0.0238 (8)	
C12	0.6283 (4)	0.6169 (3)	0.6687 (3)	0.0234 (8)	
C17	1.0179 (4)	0.7185 (3)	0.2689 (3)	0.0234 (8)	
C10	0.8047 (4)	0.6320 (3)	0.5124 (3)	0.0232 (8)	
C6	0.5350 (4)	0.4111 (3)	0.7290 (3)	0.0244 (8)	
C11	0.7204 (4)	0.6888 (3)	0.5992 (3)	0.0233 (8)	
C4	0.4350 (5)	0.0899 (3)	0.6203 (3)	0.0283 (9)	
H4A	0.5343	0.0541	0.6424	0.034*	
H4B	0.3957	0.0503	0.5495	0.034*	
C18	0.9175 (4)	0.6575 (3)	0.3365 (3)	0.0266 (8)	
H18	0.8544	0.5840	0.3091	0.032*	
C1	0.4257 (5)	0.3276 (3)	0.8895 (3)	0.0316 (9)	
H1	0.3921	0.3126	0.9563	0.038*	
C16	1.1143 (4)	0.8288 (3)	0.3098 (3)	0.0246 (8)	
C7	0.6171 (4)	0.4855 (3)	0.6544 (3)	0.0222 (8)	
C3	0.3020 (5)	0.0654 (3)	0.6958 (3)	0.0321 (9)	
H3A	0.2005	0.0975	0.6721	0.039*	
H3B	0.2766	−0.0223	0.6953	0.039*	
C2	0.4171 (4)	0.2411 (3)	0.8038 (3)	0.0267 (8)	
C9	0.7834 (4)	0.5067 (3)	0.5003 (3)	0.0271 (9)	
H9	0.8317	0.4658	0.4434	0.033*	
C15	1.1051 (4)	0.8750 (3)	0.4161 (3)	0.0269 (9)	
H15	1.1670	0.9490	0.4434	0.032*	
C20	1.1119 (5)	0.6696 (3)	−0.0180 (3)	0.0324 (9)	
H20A	1.0131	0.6106	−0.0349	0.039*	
H20B	1.2096	0.6267	−0.0301	0.039*	
C8	0.6936 (4)	0.4353 (3)	0.5676 (3)	0.0263 (8)	
H8	0.6856	0.3505	0.5529	0.032*	
C5	0.4799 (4)	0.2883 (3)	0.7122 (3)	0.0223 (8)	
C14	1.0056 (4)	0.8128 (3)	0.4820 (3)	0.0248 (8)	
H14	1.0033	0.8448	0.5532	0.030*	
C19	1.0201 (5)	0.6693 (3)	0.1565 (3)	0.0269 (9)	
O2	0.4793 (3)	0.2186 (2)	0.61587 (19)	0.0285 (6)	
C22	1.1032 (6)	0.7054 (4)	−0.2067 (3)	0.0497 (12)	
H22A	1.2023	0.6651	−0.2161	0.075*	
H22B	1.0984	0.7654	−0.2554	0.075*	
H22C	1.0059	0.6461	−0.2206	0.075*	
C21	1.1078 (5)	0.7685 (4)	−0.0920 (3)	0.0395 (10)	
C23	1.2670 (7)	0.8590 (4)	−0.0682 (4)	0.0610 (15)	
H23A	1.3636	0.8163	−0.0795	0.091*	
H23B	1.2720	0.8963	0.0046	0.091*	
H23C	1.2650	0.9213	−0.1149	0.091*	
C24	0.9523 (7)	0.8333 (5)	−0.0752 (4)	0.0657 (15)	
H24A	0.9500	0.8964	−0.1209	0.099*	
H24B	0.9544	0.8691	−0.0021	0.099*	
H24C	0.8541	0.7754	−0.0919	0.099*	
H19	0.946 (4)	0.594 (4)	0.131 (3)	0.030 (10)*	
H16	1.202 (7)	0.837 (6)	0.180 (5)	0.10 (2)*	

### Atomic displacement parameters (Å^2^)

**Table:**

	*U*^11^	*U*^22^	*U*^33^	*U*^12^	*U*^13^	*U*^23^
S1	0.0488 (6)	0.0254 (6)	0.0260 (6)	−0.0024 (5)	0.0096 (5)	0.0012 (4)
S2	0.0518 (7)	0.0221 (5)	0.0359 (6)	0.0065 (5)	0.0179 (5)	0.0056 (4)
O1	0.0512 (17)	0.0247 (15)	0.0291 (15)	−0.0056 (13)	0.0027 (13)	0.0064 (12)
O3	0.0327 (14)	0.0299 (15)	0.0296 (15)	−0.0076 (12)	0.0082 (12)	0.0044 (12)
N3	0.0350 (17)	0.0238 (17)	0.0247 (17)	0.0027 (14)	0.0082 (14)	0.0045 (14)
N2	0.0361 (18)	0.0239 (17)	0.0261 (18)	0.0049 (14)	0.0092 (14)	0.0044 (14)
N1	0.0374 (18)	0.0250 (17)	0.0292 (18)	0.0056 (14)	0.0108 (15)	0.0044 (14)
C13	0.0249 (19)	0.0203 (19)	0.027 (2)	0.0017 (15)	0.0036 (16)	0.0043 (16)
C12	0.0226 (18)	0.023 (2)	0.025 (2)	0.0034 (15)	0.0001 (16)	0.0063 (16)
C17	0.0268 (19)	0.0218 (19)	0.0216 (19)	0.0042 (15)	0.0018 (16)	0.0014 (15)
C10	0.0228 (18)	0.023 (2)	0.024 (2)	0.0024 (15)	0.0042 (15)	0.0050 (16)
C6	0.0288 (19)	0.023 (2)	0.023 (2)	0.0062 (16)	0.0048 (16)	0.0049 (15)
C11	0.0264 (19)	0.0202 (19)	0.0238 (19)	0.0038 (15)	0.0017 (16)	0.0042 (15)
C4	0.033 (2)	0.0195 (19)	0.031 (2)	−0.0030 (16)	−0.0007 (17)	0.0024 (16)
C18	0.0257 (19)	0.021 (2)	0.033 (2)	0.0017 (16)	0.0057 (17)	0.0041 (16)
C1	0.041 (2)	0.032 (2)	0.022 (2)	0.0002 (18)	0.0082 (17)	0.0071 (17)
C16	0.0185 (17)	0.028 (2)	0.029 (2)	0.0018 (15)	0.0062 (15)	0.0071 (16)
C7	0.0220 (18)	0.0224 (19)	0.0229 (19)	0.0021 (15)	0.0028 (15)	0.0056 (15)
C3	0.036 (2)	0.025 (2)	0.035 (2)	−0.0021 (17)	0.0007 (18)	0.0065 (17)
C2	0.031 (2)	0.024 (2)	0.026 (2)	−0.0004 (16)	0.0069 (16)	0.0071 (16)
C9	0.033 (2)	0.026 (2)	0.023 (2)	0.0038 (17)	0.0101 (16)	0.0018 (16)
C15	0.0237 (19)	0.023 (2)	0.032 (2)	−0.0043 (16)	0.0007 (16)	0.0013 (16)
C20	0.042 (2)	0.028 (2)	0.028 (2)	0.0037 (18)	0.0107 (18)	0.0045 (17)
C8	0.031 (2)	0.0170 (19)	0.032 (2)	0.0043 (16)	0.0066 (17)	0.0046 (16)
C5	0.0263 (18)	0.0205 (19)	0.0192 (19)	0.0022 (15)	0.0007 (15)	0.0003 (15)
C14	0.0256 (19)	0.024 (2)	0.024 (2)	−0.0002 (16)	0.0008 (16)	0.0037 (16)
C19	0.035 (2)	0.021 (2)	0.025 (2)	0.0041 (17)	0.0053 (17)	0.0015 (16)
O2	0.0390 (15)	0.0207 (13)	0.0248 (14)	−0.0011 (11)	0.0063 (12)	0.0003 (11)
C22	0.071 (3)	0.052 (3)	0.025 (2)	−0.003 (2)	0.002 (2)	0.010 (2)
C21	0.054 (3)	0.038 (3)	0.027 (2)	0.005 (2)	0.003 (2)	0.0107 (19)
C23	0.091 (4)	0.047 (3)	0.041 (3)	−0.023 (3)	0.011 (3)	0.013 (2)
C24	0.085 (4)	0.063 (4)	0.058 (3)	0.039 (3)	0.002 (3)	0.018 (3)

### Geometric parameters (Å, º)

**Table:**

S1—C1	1.710 (4)	C1—H1	0.9300
S1—C6	1.740 (4)	C16—C15	1.389 (5)
S2—N1	1.606 (3)	C7—C8	1.376 (5)
S2—N2	1.613 (3)	C3—H3A	0.9700
O1—C2	1.367 (4)	C3—H3B	0.9700
O1—C3	1.440 (5)	C2—C5	1.424 (5)
O3—C16	1.356 (4)	C9—C8	1.408 (5)
O3—H16	0.98 (6)	C9—H9	0.9300
N3—C19	1.276 (5)	C15—C14	1.380 (5)
N3—C20	1.461 (5)	C15—H15	0.9300
N2—C11	1.346 (5)	C20—C21	1.525 (5)
N1—C12	1.345 (5)	C20—H20A	0.9700
C13—C18	1.389 (5)	C20—H20B	0.9700
C13—C14	1.408 (5)	C8—H8	0.9300
C13—C10	1.468 (5)	C5—O2	1.362 (4)
C12—C7	1.437 (5)	C14—H14	0.9300
C12—C11	1.439 (5)	C19—H19	0.98 (4)
C17—C18	1.394 (5)	C22—C21	1.530 (6)
C17—C16	1.401 (5)	C22—H22A	0.9600
C17—C19	1.462 (5)	C22—H22B	0.9600
C10—C9	1.368 (5)	C22—H22C	0.9600
C10—C11	1.438 (5)	C21—C24	1.519 (6)
C6—C5	1.372 (5)	C21—C23	1.532 (6)
C6—C7	1.466 (5)	C23—H23A	0.9600
C4—O2	1.439 (4)	C23—H23B	0.9600
C4—C3	1.498 (5)	C23—H23C	0.9600
C4—H4A	0.9700	C24—H24A	0.9600
C4—H4B	0.9700	C24—H24B	0.9600
C18—H18	0.9300	C24—H24C	0.9600
C1—C2	1.351 (5)		
			
C1—S1—C6	92.66 (18)	O1—C2—C5	122.6 (3)
N1—S2—N2	100.98 (16)	C10—C9—C8	124.8 (3)
C2—O1—C3	110.9 (3)	C10—C9—H9	117.6
C16—O3—H16	102 (4)	C8—C9—H9	117.6
C19—N3—C20	119.5 (3)	C14—C15—C16	121.1 (3)
C11—N2—S2	106.5 (2)	C14—C15—H15	119.5
C12—N1—S2	106.8 (2)	C16—C15—H15	119.5
C18—C13—C14	116.9 (3)	N3—C20—C21	112.1 (3)
C18—C13—C10	120.8 (3)	N3—C20—H20A	109.2
C14—C13—C10	122.2 (3)	C21—C20—H20A	109.2
N1—C12—C7	125.8 (3)	N3—C20—H20B	109.2
N1—C12—C11	112.9 (3)	C21—C20—H20B	109.2
C7—C12—C11	121.4 (3)	H20A—C20—H20B	107.9
C18—C17—C16	118.9 (3)	C7—C8—C9	122.9 (3)
C18—C17—C19	120.4 (3)	C7—C8—H8	118.6
C16—C17—C19	120.6 (3)	C9—C8—H8	118.6
C9—C10—C11	114.4 (3)	O2—C5—C6	123.3 (3)
C9—C10—C13	122.4 (3)	O2—C5—C2	122.8 (3)
C11—C10—C13	123.2 (3)	C6—C5—C2	113.8 (3)
C5—C6—C7	127.6 (3)	C15—C14—C13	121.2 (3)
C5—C6—S1	109.2 (3)	C15—C14—H14	119.4
C7—C6—S1	123.1 (3)	C13—C14—H14	119.4
N2—C11—C10	125.8 (3)	N3—C19—C17	121.5 (3)
N2—C11—C12	112.8 (3)	N3—C19—H19	121 (2)
C10—C11—C12	121.3 (3)	C17—C19—H19	118 (2)
O2—C4—C3	112.8 (3)	C5—O2—C4	113.3 (3)
O2—C4—H4A	109.0	C21—C22—H22A	109.5
C3—C4—H4A	109.0	C21—C22—H22B	109.5
O2—C4—H4B	109.0	H22A—C22—H22B	109.5
C3—C4—H4B	109.0	C21—C22—H22C	109.5
H4A—C4—H4B	107.8	H22A—C22—H22C	109.5
C13—C18—C17	122.8 (3)	H22B—C22—H22C	109.5
C13—C18—H18	118.6	C24—C21—C20	109.2 (4)
C17—C18—H18	118.6	C24—C21—C23	110.8 (4)
C2—C1—S1	111.8 (3)	C20—C21—C23	109.3 (4)
C2—C1—H1	124.1	C24—C21—C22	110.5 (4)
S1—C1—H1	124.1	C20—C21—C22	107.6 (3)
O3—C16—C15	119.6 (3)	C23—C21—C22	109.5 (4)
O3—C16—C17	121.3 (3)	C21—C23—H23A	109.5
C15—C16—C17	119.1 (3)	C21—C23—H23B	109.5
C8—C7—C12	115.1 (3)	H23A—C23—H23B	109.5
C8—C7—C6	122.6 (3)	C21—C23—H23C	109.5
C12—C7—C6	122.2 (3)	H23A—C23—H23C	109.5
O1—C3—C4	111.3 (3)	H23B—C23—H23C	109.5
O1—C3—H3A	109.4	C21—C24—H24A	109.5
C4—C3—H3A	109.4	C21—C24—H24B	109.5
O1—C3—H3B	109.4	H24A—C24—H24B	109.5
C4—C3—H3B	109.4	C21—C24—H24C	109.5
H3A—C3—H3B	108.0	H24A—C24—H24C	109.5
C1—C2—O1	124.9 (3)	H24B—C24—H24C	109.5
C1—C2—C5	112.5 (3)		
			
N1—S2—N2—C11	−0.4 (3)	S1—C6—C7—C12	−22.0 (5)
N2—S2—N1—C12	0.2 (3)	C2—O1—C3—C4	49.4 (4)
S2—N1—C12—C7	−179.5 (3)	O2—C4—C3—O1	−59.5 (4)
S2—N1—C12—C11	0.1 (4)	S1—C1—C2—O1	−178.6 (3)
C18—C13—C10—C9	33.4 (5)	S1—C1—C2—C5	1.2 (4)
C14—C13—C10—C9	−144.0 (4)	C3—O1—C2—C1	157.4 (4)
C18—C13—C10—C11	−147.7 (3)	C3—O1—C2—C5	−22.4 (5)
C14—C13—C10—C11	35.0 (5)	C11—C10—C9—C8	−2.5 (5)
C1—S1—C6—C5	1.0 (3)	C13—C10—C9—C8	176.5 (3)
C1—S1—C6—C7	−175.1 (3)	O3—C16—C15—C14	178.8 (3)
S2—N2—C11—C10	179.5 (3)	C17—C16—C15—C14	1.3 (5)
S2—N2—C11—C12	0.5 (4)	C19—N3—C20—C21	133.7 (4)
C9—C10—C11—N2	−176.4 (3)	C12—C7—C8—C9	2.9 (5)
C13—C10—C11—N2	4.6 (6)	C6—C7—C8—C9	−175.1 (3)
C9—C10—C11—C12	2.5 (5)	C10—C9—C8—C7	−0.2 (6)
C13—C10—C11—C12	−176.5 (3)	C7—C6—C5—O2	−7.0 (6)
N1—C12—C11—N2	−0.4 (4)	S1—C6—C5—O2	177.2 (3)
C7—C12—C11—N2	179.2 (3)	C7—C6—C5—C2	175.3 (3)
N1—C12—C11—C10	−179.4 (3)	S1—C6—C5—C2	−0.5 (4)
C7—C12—C11—C10	0.2 (5)	C1—C2—C5—O2	−178.1 (3)
C14—C13—C18—C17	−0.3 (5)	O1—C2—C5—O2	1.7 (6)
C10—C13—C18—C17	−177.8 (3)	C1—C2—C5—C6	−0.4 (5)
C16—C17—C18—C13	0.5 (5)	O1—C2—C5—C6	179.4 (3)
C19—C17—C18—C13	−178.4 (3)	C16—C15—C14—C13	−1.2 (5)
C6—S1—C1—C2	−1.3 (3)	C18—C13—C14—C15	0.7 (5)
C18—C17—C16—O3	−178.4 (3)	C10—C13—C14—C15	178.1 (3)
C19—C17—C16—O3	0.5 (5)	C20—N3—C19—C17	−178.4 (3)
C18—C17—C16—C15	−0.9 (5)	C18—C17—C19—N3	−176.4 (3)
C19—C17—C16—C15	177.9 (3)	C16—C17—C19—N3	4.7 (5)
N1—C12—C7—C8	176.7 (3)	C6—C5—O2—C4	173.0 (3)
C11—C12—C7—C8	−2.8 (5)	C2—C5—O2—C4	−9.5 (5)
N1—C12—C7—C6	−5.3 (5)	C3—C4—O2—C5	37.3 (4)
C11—C12—C7—C6	175.2 (3)	N3—C20—C21—C24	−60.4 (5)
C5—C6—C7—C8	−19.5 (6)	N3—C20—C21—C23	61.0 (5)
S1—C6—C7—C8	155.9 (3)	N3—C20—C21—C22	179.7 (3)
C5—C6—C7—C12	162.7 (3)		

### Hydrogen-bond geometry (Å, º)

**Table:**

*D*—H···*A*	*D*—H	H···*A*	*D*···*A*	*D*—H···*A*
O3—H16···N3	0.98 (6)	1.64 (7)	2.569 (4)	155 (6)
C4—H4*A*···O3^i^	0.97	2.39	3.200 (5)	140

Symmetry code: (i) −*x*+2, −*y*+1, −*z*+1.
